# Shedding light on the etiology of neurodegenerative diseases and dementia: the exposome paradigm

**DOI:** 10.1038/s44184-022-00018-3

**Published:** 2022-11-16

**Authors:** Fabio Cavaliere, Sinan Gülöksüz

**Affiliations:** 1 Achucarro Basque Center for Neuroscience, Leioa, Spain; 2 Instituto Biofisika (UPV/EHU, CSIC), Leioa, Spain; 3 Department of Psychiatry and Neuropsychology, School for Mental Health and Neuroscience, Maastricht University Medical Center, Maastricht, the Netherlands; 4 Department of Psychiatry, Yale University School of Medicine, New Haven, CT USA

**Keywords:** Neuroscience, Health care, Disease prevention

## Abstract

Age is the main risk factor of neurodegenerative diseases, but environmental exposure and lifestyle are important candidates for understanding their etiology. Accumulating evidence suggests that “*exposome”*, described as the totality of human environmental exposures from conception onwards, represents major modifiable risk factors for most neurodegenerative diseases and dementia. In this commentary, we discuss and provide our opinion about the urgent need for a constructive dialog between political stakeholders, researchers, and physicians to implement specific strategies to counteract and reduce the onset of neurodegenerative diseases.

The increased number of neurodegenerative diseases and other forms of dementia is becoming a worldwide emergency, with a significant impact on public health, economy, and social wellness. Around 50 million people worldwide suffer from dementia, and the number of people with dementia is set to triple by 2050^1^.

The WHO guidelines on risk reduction of dementia^1^ represent an important source for health care providers, governments, policy-makers, and other stakeholders, to prevent the increasing number of neurodegenerative diseases and dementia. According to these guidelines, it is imperative for policy makers to identify and reduce modifiable non-genetic risk factors to pave the way for a healthy lifestyle to delay brain aging. Meta-analytical research, combined with database analysis, has thus far identified at least 12 largely modifiable high-risk environmental factors over the entire life of an individual: low educational level during early life; hearing loss, excessive alcohol consumption, hypertension, traumatic brain injuries, smoking, obesity during the midlife; social isolation, physical inactivity, depression, diabetes, and air pollution during the late life^2^ (Fig. 1). Prevention of these risk factors might reduce at least 40% of dementia cases^2^.

**Fig. 1:**
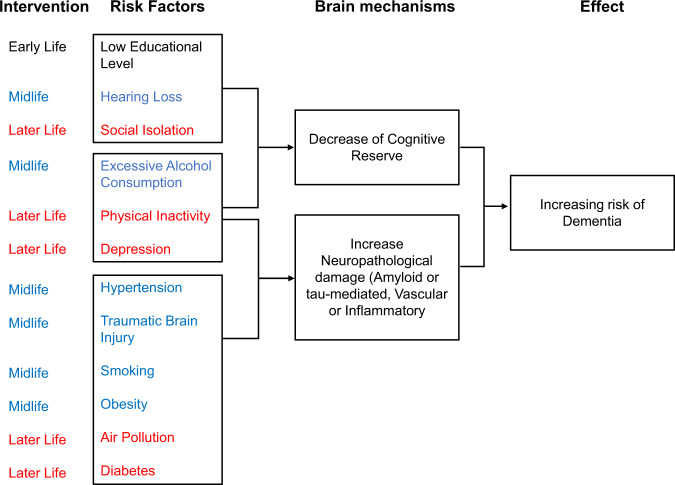
Twelve non-genetic risk factors and possible mechanisms leading to dementia. Modified from Livingston et al. ^2^.

The term “environment” includes different factors, such as diet, pathogenic infection, drug use, and psychological stressors^3^. The combination of all environmental exposures, including pollutants, lifestyle factors and behaviors, over the lifespan is defined as “*exposome*”. Air pollution and the continued use of pesticides are strongly associated with neurodegeneration in several chronic diseases^4^. Prolonged exposures to particulate matter or nanoparticles, generated by incomplete combustion of fuel used for transportation or industry, lead to respiratory tract inflammation and microglial activation in the CNS^5^. Due to their microscopic size, these particles can easily reach the peripheral circulatory system and the brain, where they can induce inflammatory reactions and give rise to the production of reactive oxygen species^6^. The presence of some of these contaminants in the postmortem brain has been linked to Aβ plaques in Alzheimer’s disease (AD) and Lewy bodies in Parkinson’s disease (PD)^7,8^. Pesticides like organochlorine compounds are lipophilic, easily absorbed, and stored in body fat and lipid-rich tissues for long periods^9^. A pioneering study by Calderón-Garcidueñas et al. showed atypical dementia in dogs of Mexico City^10^ suggesting a direct role of pollutants in activating neurodegeneration in dogs’ brains. Chronic exposure to pollutants and pesticides has also been associated with the presence of Lewy bodies in human postmortem brains^11^. Recently, an electron holography study revealed that AD patients showed a massive increase of magnetite nanospheres, especially surrounding the β-amyloid plaques^12^. The magnetite nanospheres are particular matters lower than PM2.5 that are formed by the combustion and/or friction-derived heating of car brakes, specifically and highly present in urban areas^13^. More recently, Calderón-Garcidueñas et al. described the presence of aberrant AD and PD markers, as well as solid metal nanoparticles in the brains of children and young adults exposed to Metropolitan Mexico City pollution^14^. Similarly, high concentrations of these heavy metals (iron and manganese) detected in air pollution may generate cell degeneration in the basal ganglia circuitry^15^. Unexpectedly, the toxic impact of metals on the brain may occur not only later in life (e.g. through occupational exposures in the steel industry, battery production, or as a consequence of an intravenous injection of illicit drugs) but also during childhood, with detrimental consequences in adult life.

The diversity of risk factors poses a major challenge for uncovering the etiology of neurodegenerative diseases. Although some of these risk factors may initially be considered behavioral patterns rather than environmental factors, it is often difficult to draw a line between environment and behavior that often interact and influence each other dynamically. In this regard, our environment is also a significant factor that shapes our behaviors, which may subsequently influence our environment. Furthermore, policies have a major impact on our environment and behavioral patterns. For instance, constructing urban bicycle roads and disincentivizing private car use would increase the number of cyclists, improve physical activity levels, and reduce the prevalence of obesity. Additionally, reducing private transportation but increasing public transportation would decrease air pollution levels.

The exposome paradigm may help in explaining the main cellular characteristics of dementia and other neurodegenerative diseases: chronic and multifactorial nature. First proposed by a cancer epidemiologist, Chris Wild^16^, later refined by others like Gary Miller^17^ and Rappaport and Smith^18^, the exposome framework provides a holistic view of environmental etiology. Especially, Rappaport and Smith extended the definition of environmental exposure by including not only the chemicals ingested from food, air, or water but also the internal environment, such as chemicals produced by inflammation, oxidative stress, and lipid peroxidation.

There is a growing investment in exposome research worldwide. By acknowledging the urgency for studying the non-genetic/environmental factors involved in the etiology of chronic diseases, the National Institute of Environmental Health Sciences defined exposome research as a major goal of its 2018–2023 strategic plan and funded several projects, such as the Health and Exposome Research Center (HERCULES) and the Children’s Health Exposure Analysis Resource (CHEAR). Similarly, the European Commission (EC) funded the European Human Exposome Network (https://www.humanexposome.eu/). In the last five years, the EC invested 106 million euros in research and innovation projects related to human exposome in immune-mediated diseases, metabolism, mental health, and pulmonary diseases. Nevertheless, none of these funded projects specifically addresses neurodegenerative diseases. Because of the involvement of multiple factors in the onset and progression of neurodegeneration, the exposome research in neurodegenerative diseases is challenging and requires an interdisciplinary approach: exposomics. The exposomics approach relies on the application of multi-*omics* techniques, chemo-informatics, reliable human bio-models, the analysis of large cohorts, and often the use of methodologies that produce massive amounts of data, thus requiring “Big Data” processing. Clearly, this high-level interdisciplinarity can only be achieved through large consortiums.

## Proposed strategies to reduce the risk of neurodegenerative diseases

1. *Funding Multidisciplinary research*. According to the most recent WHO Health and Climate Change Survey Report, only 12 out of 101 countries have developed a national curriculum to train health personnel on the health impacts of environmental contamination and climate change. In combination with a “green policy”, funding research to reduce environmental contamination through the work of multidisciplinary consortia will contribute to targeting the diverse range of exposures that can impact brain health, as well as better defining the etiology of neurodegenerative diseases. A multidisciplinary approach is needed to better understand the multifactorial etiology of neurodegenerative diseases and achieve actionable insights for policy-making.
2. *Reducing the exposure to contamination*. It is important to identify and characterize known and unknown chemicals linked to neurodegenerative diseases. In this field, applying the novel exposomics, such as cheminformatics and ultrahigh resolution mass spectrometers, would be beneficial. Efforts to establish policies to reduce population exposure to air pollution should be accompanied by efforts to identify early pathways and biomarkers of neurodegeneration to act promptly.
3. *Promoting physical activity and increasing the quality of life style*. Promoting physical activity and reducing obesity will have additional positive effects on diabetes, which is also a specific risk factor for AD. Policies must be directed to improve healthy food availability and design an environment to increase movement, especially at midlife and on.

## Conclusion

It is time to gain further insight into *when*, *how*, and *why* neurodegenerative diseases begin. A longer life expectancy, together with an environment altered by pollution, sedentary lifestyle, financial burden, and psychological stress, has given rise to an emergency with massive economic and societal costs. Public stakeholders, researchers, and physicians should consider dementia and chronic neurodegeneration as a combination of alterations and act together to tackle neurodegenerative diseases. Significant progress has been made in the field of environmental contamination and its correlation with neurodegenerative diseases. To dissect causal links from environmental correlates, we need to understand the molecular and biochemical basis that governs the onset of neurodegeneration and characterize early biological markers. These efforts require the urgent collaboration of different disciplines. Basic scientists, clinicians, epidemiologists, data scientists, and key stakeholders can contribute together to encompass the high-risk factors described above and shed light on the etiology of neurodegenerative diseases and dementia. In this regard, the exposome framework can provide the path to success in this endeavor.
